# The Evaluation of the Balance Test and MuscleLab Test for the Early Detection of Femoroacetabular Impingement

**DOI:** 10.3390/jfmk8040159

**Published:** 2023-11-14

**Authors:** Roberto Centemeri, Michele Augusto Riva, Michael Belingheri, Maria Emilia Paladino, Marco Italo D’Orso, Jari Intra

**Affiliations:** 1 Department of Occupational Health, University of Milano-Bicocca, Fondazione IRCCS San Gerardo dei Tintori, 20900 Monza, Italy; roberto.centemeri@unimib.it (R.C.); michele.riva@unimib.it (M.A.R.); michael.belingheri@unimib.it (M.B.); maria.paladino@unimib.it (M.E.P.); marco.dorso@unimib.it (M.I.D.); 2 Clinical Chemistry Laboratory, Fondazione IRCCS San Gerardo dei Tintori, 20900 Monza, Italy

**Keywords:** femoroacetabular impingement, altered hip joint, FABER, FADIR, functional test, neural–musculoskeletal system

## Abstract

Femoroacetabular impingement (FAI) is a common source of hip pain affecting a wide range of subjects. In this work, we assessed two tests, namely the balance test and the MuscleLab test, in comparison with the flexion–abduction–external rotation (FABER) and flexion–adduction–internal rotation (FADIR) tests, in order to evaluate the functionality of the neural–musculoskeletal system of the subjects affected by FAI based on the measurement of biomechanical parameters. Our goal was to investigate the early detection of an altered hip joint and to monitor pathology progression over time. A total of 52 subjects, 29 females and 23 males, with an average age of 42 ± 13 years presenting hip impingement diagnosed using X-ray, were enrolled. Twenty-eight patients without signs of hip impingements were used as the control group. The balance test, which evaluates the capacity of a person to keep the orthostatic position against terrestrial gravity, and the MuscleLab test, which measures the force and power generated by active muscles during a movement, as well as FABER and FADIR tests, were performed in each subject. The balance and MuscleLab tests presented 100% sensitivity and higher sensitivity in FAI diagnosis, with 72.9% and 70.4%, respectively, in comparison with those obtained using FABER and FADIR tests, with 59.6% and 67.3%, respectively. The evaluation of the neural–musculoskeletal system using the balance and MuscleLab tests can help in the early detection of the severity of hip impingements and the assessment of non-operative treatments used over time.

## 1. Introduction

Femoroacetabular impingement (FAI), defined as a pathological condition for the first time by Ganz and co-authors in 2003, is considered a common cause of chronic hip pain, decreased function, and progression to early osteoarthritis, resulting from morphological abnormalities of the proximal femur and/or acetabulum [1,2,3]. In 2016, the Warwick Agreement consensus statement defined the concept of FAI syndrome (FAIS), which consists of the triad of symptoms, clinical signs, and radiographic osseous changes [4,5,6]. Flexion–abduction–external rotation (FABER) and flexion–adduction–internal rotation (FADIR) tests are two key physical examinations used to identify the particular causes of pain, while hip radiographs identify the specific bony deformities [7]. The most common osseous abnormalities predisposing to FAI include cam or pincer lesions: (I) Cam-type impingement, which is characterised by decreased head–neck offset or the abnormally shaped femoral head, and (II) pincer-type impingement, which is characterised by an excess coverage of the acetabulum over the femoral head. These structural lesions can occur alone or in combination (mixed), with the latter considered the most common diagnosis. The severity of osseous deformity is measured by the alpha and lateral central-edge calculated angles for cam- and pincer-impingements, respectively. FAI predominately affects physically active young and adult people, and in particular, athletes, among whom cam morphology is more present, from two to eight times, compared with the general population [1,2,3,4,5,6]. Moreover, FAI is a common source of hip pain in children and adolescents, and recent studies demonstrated the effects of sex, race, and sports on FAI development in the paediatric population [6,7,8,9]. Management strategies include first-line conservative options, including pharmacological and non-pharmacological options, such as muscular training and activity modification; osteopathic manipulative treatments, which facilitate a reduction in impingement and an increase in the range of motion; or surgical treatment when severe symptoms are present and conservative therapy fails [1,2,3,4,5,6].

Currently, the examination of the affected hip requires passive manipulation, but it has been demonstrated that in subjects affected by FAI, adaptive changes are used to avoid pain or frequent symptoms [2,10]. Commonly, balance and muscle tests are performed to assess and monitor health status and functional performance over time, particularly in older adults [11,12]. Several studies have demonstrated that decreased balance and muscle strength are associated with early age-related decline [11,12]. Therefore, in our study, we used the balance test and MuscleLab test to evaluate the functionality of the neural–musculoskeletal system of subjects affected by FAI by measuring biomechanical parameters, in order to improve the early detection of an altered hip joint compared with the standard FABER and FADIR tests, and to monitor pathology progression over time.

## 2. Materials and Methods

### 2.1. Patients and Study Design

Fifty-two patients with symptoms of femoroacetabular impingement (FAI) assessed by a single physician (R.C.) specialised in posturology, between May 2018 and April 2019, presented to the IRCCS San Gerardo dei Tintori for a medical examination. Baseline characteristics were recorded, including age, sex, anatomical regions of hip pain, and type of occupation. The diagnosis of FAI was made on clinical and radiographic grounds. Radiological data consisted of different types of views, such as an anteroposterior view of the pelvis, a cross-table lateral view, a 45° or 90° Dunn view, a frog-leg lateral view, and a false-profile view, as recommended by the Academic Network for Conservational Hip Outcomes Research (ANCHOR) study group [10]. The physical examination of the hip included inspection, palpation, and the evaluation of the range of motion using the flexion–abduction–external rotation (FABER) and flexion–adduction–internal rotation (FADIR) tests, which are both commonly utilised for the diagnosis of FAI [3]. It has been well documented that FAI is the most common cause of labral tears, which present the same patterns of pain and symptoms. The anterior hip impingement test was performed by provoking pain with flexion, adduction, and internal rotation and was used to exclude labral lesions in all the subjects enrolled, as previously reported [13,14]. Twenty-eight patients without signs of hip impingement were considered the control group.

All the subjects were assessed using FADER, FABER, Balance test, and MuscleLab test, in order to examine the neural-musculoskeletal system functionality in the presence or the absence of a hip impingement.

Ethical approval was waived by the local Ethics Committee (Fondazione IRCCS San Gerardo dei Tintori) in view of the retrospective nature of the study and de-identified data. The local ethics committee did not require informed consent because all subjects’ data were de-identified.

### 2.2. Functional Tests

#### 2.2.1. Balance Test

This test is used to analyse the orthostatic posture of patients by measuring the movements of the centre of pressure, with the purpose of assessing the ability of a person to keep the orthostatic position against terrestrial gravity and evaluating how the neural–musculoskeletal system responds to the swinging movements of the centre of pressure due to the gravity that tends to cause a person to fall forward. Falling is avoided by the activation of the muscles of the legs that interfere with the centre of pressure-swinging paths. The test was performed using a platform that allows for the measurement of the body stability, in other words, the ability of the body to return to the equilibrium state after a modification, and following the “norms 85” of the Association Posturologie Internationale (API) [15]. The model of the balance platform used was SVeP 96-6 Politecnica (Modena, Italy), with a recording frequency of 5 Hz and using SVEP 6.30 software (version 1.0, accessed on 1 December 2022: https://www.politecnica.it/stabilometria/). The records were executed in five modalities: (I) open eyes; (II) closed eyes; (III) retroflex—neck flexed backward; (IV) open eyes with insole; and (V) closed eyes with insole. The insoles were fabricated of thermoplastic material without correction of deformity, with a thickness of 0.5 mm. Every test took 51.2 s, a sufficient time to obtain data and not too long to induce fatigue. Briefly, the patient’s feet were positioned on the platform, keeping the heels distant 2 cm from each other and 30° opened. Then, the patient was asked to (I) stare at the computer screen that showed a vertical line at the level of the viewer’s eyes; (II) be relaxed; (III) keep the arms relaxed and close to the hips; and (IV) count slowly with high voice. For each test, the posturography, which involves a graphic representation of the consecutive pressure centre’s positions with respect to the support polygon’s centre of gravity, and the stabilogram, which is the graphic representation of the pressure centre’s positions in the function of time on the X axis (right–left movements), and on the Y axis (forward–backward movements), were obtained for each patient. The parameters considered were (I) average X—the average value representative of the movements along the X axis compared with the balance test’s polygon of support; (II) average Y—the average value representative of the movements along the Y axis compared with the balance test’s polygon of support; (III) surface—the ellipse that includes the 90% of the pressure centre positions recorded; (IV) LFS—the length of the pressure centre’s path during the record compared with the surface unit; and (V) VFY—the correlation between the pressure centre’s movements on the Y axis and variation in the velocity of the pressure centre’s movements. The balance test was considered positive if one parameter was out of the normal range.

#### 2.2.2. MuscleLab Test

The MuscleLab test was performed using surface electromyography with a linear encoder, which allowed for the measurement of the force and power generated by active muscles during a movement versus time. The MuscleLab’s unit (Politecnica, Modena, Italy) provided inputs for EMG sensors (4000e only) and angle sensors and was connected to a computer with the MuscleLab’s software installed on it (version 7.18). The electrodes were model T916 (Teardrop shape, 43 × 45 mm size, and 4 cm of interelectrode distance) manufactured by Bio Protech Inc. (Wonju City, Gangwondo, Republic of Korea), and hydrogel was used for the adhesion of electrodes to the skin. Briefly, the electrodes were positioned on the rectus femoris and vastus medialis, following the recommendations for sensor placement of Surface Electromyography for the Non-Invasive Assessment of Muscles (SENIAM) project. The test consisted of repeating a movement where the patient had to lift one leg and touch the step high about 40 cm with the foot; afterwards, the patient had to bring their foot back to the initial position and do the same with the opposite leg for 27 s at the frequency preferred by the patient. The test was performed in different modalities: (I) straight head, electrodes active in both legs and the encoder wrapped around the left leg; (II) head turn right, electrodes active in the left leg and the encoder wrapped around the left leg; (III) straight head, electrodes active in both legs and the encoder wrapped around the right leg; and (IV) head turn left, electrodes active in the right leg and the encoder wrapped around the right leg. The results obtained from the instrument showed the standard deviations of force and power values of each cycle movement, namely the lifting movement of the leg and the movement of bringing the leg back to the initial position. The standard deviation values of force and power were obtained considering a fixed mass of 5 kg. Finally, the physician compared the standard deviations of force and power values of the first and third modalities. The test was considered positive when force and/or power values evaluated on the left leg were greater than 10% compared with the values obtained with the right leg. Similarly, the physician also compared the force and power values of the second and fourth modalities. Regarding the last two comparisons, it is important to note that, in physiological conditions, during a movement, the rotation of the head to one side causes an increase in the force in the contralateral side’s muscles. The test was considered positive if, when turning the head to the right, the parameters of force and/or power measured on the left leg were decreased by more than 10% compared with the ones obtained when the head was straight, and/or if, when turning the head to left, the parameters of force and/or power on the right leg were decreased by more than 10% compared with the ones with the straight head.

### 2.3. Statistical Analysis

Statistical analyses were performed using MedCalc for Windows, version 19.4 (MedCalc Software, Ostend, Belgium). For each physical test, diagnostic sensitivity and diagnostic specificity were calculated. The chi-square (χ^2^) test was used to compare the results of the different physical examinations performed. A *p* value < 0.05 was considered statistically significant.

## 3. Results

### 3.1. Patients’ Baseline Characteristics

A total of 80 patients were evaluated for hip pain (Table 1). This group was composed of 45 females (56%) and 35 males (44%), with an average age of 42 ± 13 years. Radiographic examination and clinical evaluation identified the presence of femoroacetabular impingements in 52 subjects and the absence in 28, which were considered the control group. The positive subjects were separated into two groups: those with unilateral hip malformation and those with bilateral hip malformation. Out of the 52 patients, 39 (75%) were affected by hip malformation, with 32 patients (82%) having cam lesions, 2 (5%) having pincer lesions, and 5 (13%) having mixed-type impingements. Most of the patients with cam lesions had bilateral malformations (22/32, 69%), and this was more frequent in females (13/22, 59%) than in males (9/17, 53%). On the other hand, thirteen patients (25%) were affected by two different types of malformation: eight subjects showed cam lesions in one hip and mixed-type impingements in the opposite hip, four patients presented pincer lesions in one hip and mixed-type impingements in the opposite hip, and only one patient exhibited cam lesion in one hip and pincer lesion in the other.

Considering patient age, most hip impingement symptoms were observed in males aged between 30 and 41 years and in females aged between 42 and 53 years. All patients were asked to indicate if they had pain in one or more different anatomical regions, with the purpose of investigating the frequency of the pain location and the association with hip impingement. Out of the 52 patients, 22 (42.3%) reported pain in one anatomical region. The lumbar pelvic was reported as the most frequent localisation (9), followed by the knee (4), groin and cervical shoulder (3), foot (2), and thigh (1). On the other hand, 20 (38.5%) patients presented pain in two anatomical regions, namely the lumbar pelvic region and the cervical shoulder region (14/20, 70%). Ten (19.2%) patients reported pain in three or more different anatomical regions: Four patients reported pain along the lower limb, and six patients had pain at the lumbar pelvic region associated with pain to other different regions, such as knee and foot, cervical shoulder and knee, and thigh and groin. Concerning the anatomical region of pain, in the control group, similar results were observed.

Lastly, each patient was asked to indicate what kind of job they have had throughout their life, particularly in terms of whether they had only one type of occupation or more than two types. Out of the 52 patients, 38 (73.1%) had one type of occupation, and the most frequent occupation was computer terminal work (10), followed by manual handling without loads (7), manual handling with loads (6), housewife, and student (3). The other 14 (26.9%) patients had two types of occupation during their life, with manual handling with loads (8) and sitting (7) reported as the two most frequent typologies in the different combinations. In the control group, most of the subjects (*n* = 18, 64%) worked at computer terminals.

### 3.2. FABER and FADIR Tests

All 80 patients were subjected to the flexion–abduction–external rotation (FABER) and flexion–adduction–internal rotation (FADIR) tests in order to identify labral pathology and intra-articular causes of hip pain. The control group had negative results for the FABER test, whereas the results of this test for the 52 subjects affected by femoroacetabular impingements were positive in 31 subjects and negative in 21, corresponding to a sensitivity of 59.6%. The results of the FADIR test were positive in 35 subjects and negative in 17, corresponding to a sensitivity of 67.3%. Then, the correspondence between the positivity of the FABER and FADIR tests and the anatomical localisation of the hip impingement obtained via radiography was studied. The results indicated that 6 patients had a hip malformation in the left hip, 5 in the right hip, and 41 in both hips. Considering the patients with positive X-ray results in both hips, the FABER and FADIR sensitivity rates were 10% (4/41) and 12.2% (5/41), respectively. These results indicate that these two tests have low sensitivity in patients affected by hip malformation at both hips.

### 3.3. Balance and Muscle Tests

Afterwards, the balance test was performed on all patients and the results were negative in the control group, while, among the 52 subjects, excluding 4 subjects who did not complete the test, 35 tested positive, and 13 were negative (without parameters out of the normal ranges). The balance test’s sensitivity was 72.9% (35/48). Considering the different types of tests used to assess the balance system, among the patients who tested positive, the most frequent test with positive results was retroflex (80%, 28/35), followed by closed eyes (57.1%, 20/35), open eyes with the insole (57.1%, 20/35), closed eyes with the insole (54.3%, 19/35), and open eyes (51.4%, 18/35). Among the 35 patients with positive results, 19 also tested positive in both FABER and FADIR tests, and, considering these three tests simultaneously, the sensitivity for hip impingement diagnosis was 39.6% (19/48). On the other hand, if we consider the positive results of the balance test together with the positive results of the FABER and/or FADIR tests, the sensitivity increased to 58.3%. Then, the MuscleLab test was carried out on 27 subjects who tested positive, since 25 patients did not complete it, and on the control group comprising all the individuals with negative results. Considering the fact that a patient tested positive if at least one parameter was altered (see Section 2), out of the 27 patients, 19 had positive results, and 8 patients were negative. Therefore, the sensitivity of the MuscleLab test was 70.4%. Moreover, the site of hip impingement detected using X-ray was compared with the results obtained using the MuscleLab test in order to assess the performance of this dynamic approach. Three patients had a hip impingement affecting the left leg, and the results of the MuscleLab for the left leg were positive in two patients, whereas the diagnoses of two patients with hip impingement affecting the right leg were confirmed with the MuscleLab test. In the 14 patients affected by both hip impingements, the MuscleLab test had positive results in both hips in 9 patients (64.3%, 9/14), while it had positive results only in one hip in the other 5 subjects.

Taken together, the diagnostic sensitivity of the balance and MuscleLab tests, 72.9% and 70.4%, respectively, were significantly higher than those obtained using FABER and FADIR tests, 59.6% and 67.3% (*p* < 0.01).

## 4. Discussion

In our work, for the first time, we conducted an assessment of the neural–musculoskeletal system in the presence of hip impingements, to provide evidence that the monitoring of this system facilitates the early detection of the symptoms associated with FAI. Improvements in the diagnosis and treatment of hip pathology increase the need for a better understanding of the impact that pathologies may have on hip biomechanics as well as on hip function. Orthopaedics often measure biomechanical parameters such as joint motion and muscle force in order to evaluate if impairment exists [16,17]. On the other hand, radiological tests reveal radiographic changes associated with FAI and not symptoms. Therefore, we used the balance and MuscleLab tests on subjects affected by hip impingements, and we compared their findings based on the measurement of biomechanical parameters with the data obtained using standard FABER and FADIR tests. These tests are commonly used to identify most types of hip pathology, including FAI, but they have shown high variability in sensitivity and specificity values and are subject to limitations due to the ability and skill of operators who perform these clinical examinations [1,2,3].

Our study highlighted that the Balance and MuscleLab tests presented 100% specificity and higher sensitivity, 72.9 and 70.4%, respectively, in comparison with those of FABER and FADIR tests, 59.6 and 67.3%, respectively. Moreover, considering the MuscleLab test, it is interesting to observe that its sensitivity for the detection of the anatomical side of hip impingement was 81.2%, while FABER and FADIR tests presented a low level of correspondence between positive results and the site of hip malformation, with 10% and 12.2%, respectively. Other tests for FAI diagnosis have been introduced, and among them, the dynamic internal rotatory impingement (DIRI) and the dynamic external rotatory impingement (DEXRIT) tests were reported to have sensitivities of 50% and 62%, respectively, although no studies demonstrated their diagnostic accuracy in the detection of an affected hip [2,3]. Moreover, in 2018, a novel diagnostic foot progression angle walking (FPAW) test was implemented for identifying FAI or hip instability, and it was compared with FABER and FADIR tests. Ranawat and co-authors found that FPAW combined with FABER and FADIR examinations improved clinical accuracy for FAI and hip instability, and the test presented greater specificity for FAI than FADIR and had similar results to those obtained with FABER [18].

The balance and MuscleLab tests are used to assess the musculoskeletal system, not only in older adults but also in athletes and physically active people, as previously reported [11,19]. Our results demonstrated that the most sensitive test that indicated muscle–skeletal altered functionality in a subject affected by hip impingement was the MuscleLab test, where the protocols performed in our study were helpful in the diagnosis of FAI. Moreover, the most important consideration was the possibility of monitoring a subject potentially exposed to the risk of lesions of the hip over time, observing the appearance of muscle–skeletal dysfunctions. In the meantime, these tests might be helpful in the assessment of therapeutic efficacy.

The management of FAI can be performed using a non-operative treatment modality or surgery, which is used to eliminate the pathologic contact between the proximal femur and the acetabular rim. Conservative treatment is the first-line choice, and physical therapy, muscle flexibility, patient education, stretching and strengthening, and neuromuscular education are recommended, as indicated by the American Physical Therapy Association [2,3,20,21]. Also, the use of oral anti-inflammatory drugs, corticosteroids, and hyaluronic acid has shown clinically significant improvements and reduced pain [2,3]. More recently, it has been demonstrated that patients affected by the symptomatic borderline dysplasia of the hip (BHD), which may lead to FAI, can benefit from arthroscopic treatment for the stabilisation and strengthening of the joint [22,23]. On the other hand, monitoring the musculoskeletal system using the balance and MuscleLab tests allows for the early detection of symptoms associated with FAI without exposing patients to more dangerous or invasive instrumental techniques, such as radiological investigations.

Our work is a single-centre study, which is a limitation, and a larger number of individuals and further clinical studies are needed in order to verify and improve the accuracy of our results.

## 5. Conclusions

Collectively, our data highlight the effectiveness of these tests, particularly MuscleLab, in providing a better understanding of the impact of altered biomechanical parameters on subjects with structural hip pathologies. Longer-term follow-up and further studies, including, for example, single-leg stance and step-up tests, might help clinicians to perform evidence-based treatments, thus improving patients’ outcomes.

## Figures and Tables

**Table 1:** Demographics and baseline characteristics of the subjects affected by hip impingement.

Sex, female/male, *n*		29/23
Age (years, mean ± SD)		42 ± 13
Radiography		
	Positive	52
	Negative	0
Type of impingement		
	Cam	32
	Pincer	2
	Mixed-type *	18

* In this group, there are subjects with one type of impingement in both hips and subjects with two types of impingement that differ between the two hips (cam or pincer or mixed-type impingement in one hip and cam or pincer or mixed-type impingement in the other hip).

## Data Availability

The data presented in this study are available on request from the corresponding author.
